# The pH signaling pathway Pal/PacC regulates fungal growth, stress responses, and mycotoxin biosynthesis in *Fusarium graminearum*

**DOI:** 10.1007/s44297-025-00054-3

**Published:** 2025-08-01

**Authors:** Yujie Wang, Tong Cao, Dekun Liu, Hanxi Zhao, Yuan Chen, Shuolong Li, Ke Wen, Qurban Ali, Hai Huang, Shuai Zhou, Huijun Wu, Xuewen Gao, Jun Tian, Qin Gu

**Affiliations:** 1 JSNU-UWM International Cooperation Joint Research Laboratory of Food Safety and Microbial Functional Genomics, School of Life Science, Jiangsu Normal University, Xuzhou , 221116 Jiangsu, People’s Republic of China; 2 Department of Plant Pathology, College of Plant Protection, Key Laboratory of Monitoring and Management of Crop Diseases and Pest Insects, Ministry of Education, Nanjing Agricultural University, Nanjing, China; 3 Department of Clinical Laboratory, Children’s Hospital of Nanjing Medical University, Nanjing, China; 4 Department of Biology, College of Science, United Arab Emirates University, Al-Ain, Abu-Dhabi, United Arab Emirates

**Keywords:** *Fusarium graminearum*, Pal/PacC pathway, Interaction, Biological function, DON biosynthesis

## Abstract

The Pal/PacC pathway, which regulates genes that adapt to alterations in ambient pH, has been extensively studied in *Aspergillus nidulans*. Currently, the functions of the Pal/PacC pathway in *Fusarium graminearum* are poorly understood. In this study, we constructed targeted deletions of Pal/PacC pathway components through homologous recombination, including FgPalA, FgPalB, FgPalC, FgPalF, FgPalH, FgPalI and the pH-responsive transcription factor FgPacC. The phenotypic assay results indicated that the aerial hyphae of the ΔFgPalA, ΔFgPalB, ΔFgPalC, ΔFgPalF, ΔFgPalH and ΔFgPacC mutants of *F. graminearum* were significantly decreased in the PDA, MM and CM media and exhibited increased sensitivity to various environmental stresses. In addition, the western blot analysis revealed that the key element FgPacC could be cleaved to the functional isoform FgPacC30 after being subjected to alkaline pH or, independently, NaCl-induced osmotic stress. Further studies elucidated the direct interactions between the essential components of this signaling pathway in *F. graminearum* for the first time and revealed that ambient-pH-dependent proteolytic activation regulates the subcellular localization of FgPacC. More importantly, we revealed that FgPacC30 inhibits the activity of the histone acetyltransferase FgGcn5, thus transcriptionally down-regulating the expression of the *FgTRI1* gene and inhibiting DON biosynthesis. Overall, our study suggests that the pH regulatory system plays a critical role in the response to external stress and virulence in *F. graminearum*, providing a new perspective for understanding the pathogenic mechanisms in phytopathogenic fungi.

**Supplementary Information:**

The online version contains supplementary material available at 10.1007/s44297-025-00054-3.

## Introduction

Pathogenic fungi have developed intricate mechanisms to cope with and adapt to dynamic environmental changes to ensure their survival, proliferation and colonization [1, 2]. The ambient pH serves as a pivotal signal that regulates numerous cellular processes, including growth, cell wall and membrane integrity, protein stability, and secondary metabolism [3–6]. Pathogenic fungi perceive pH signals and regulate gene expression to change or adapt to the ambient pH, thereby facilitating fungal pathogenicity and infection [7]. For example, *Penicillium* spp., *Botrytis cinerea*, and *Sclerotinia sclerotiorum* can secrete organic acids to acidify the external environment [8–10]. *Alternaria alternata* and *Colletotrichum* spp. drive fungal pathogenicity by releasing ammonia to alkalize host tissues [11]. Moreover, *Fusarium oxysporum* and *F. graminearum* trigger host alkalinization by secreting a functional homologue of rapid alkalinizing factor (RALF), thereby increasing fungal infection potential and suppressing host immunity [12, 13]. These findings emphasise the critical role of ambient pH regulatory mechanisms in fungal pathogen growth and virulence and provide insights into the survival strategies of pathogens and the mechanisms of interactions with host plants.

The Pal/PacC regulatory pathway of pathogenic fungi in response to pH signals has been well studied in *Saccharomyces cerevisiae* and *Aspergillus nidulans* [2, 14–16]. The Pal/PacC signaling pathway typically consists of six Pal proteins (PalA/Rim20, PalB/Rim13, PalC/Rim23, PalF/Rim8, PalH/Rim21, and PalI/Rim9), along with the crucial zinc-finger transcription factor PacC/Rim101 and multiple endocytic machinery components [2, 15]. In *A. nidulans*, PacC72 (72-kDa), the full-length form, undergoes two successive proteolytic cleavages to generate PacC53 (53-kDa) and PacC27 (27 kDa) [2]. Under acidic conditions, PacC72 adopts a closed conformation that is inaccessible to processing proteases and is located in the cytoplasm. However, the situation is entirely distinct under alkaline conditions. First, the transmembrane sensor PalH detects alkaline pH and triggers PalF ubiquitination and phosphorylation, which promotes endocytosis of the receptor complex and recruits the endosomal sorting complexes required for transport (ESCRT) to the plasma membrane. The PalA-PalB-PalC-associated proteasome performs pH-dependent cleavage of PacC72, removing its inhibitory C-terminal domain to generate the intermediate PacC53 [17, 18]. Subsequently, PacC53 undergoes additional proteasomal processing in a pH-independent manner, resulting in mature and functional PacC27. This truncated form of PacC27 then enters the nucleus, where it promotes the expression of alkaline-responsive genes while suppressing those induced under acidic conditions [2, 5, 19]. More importantly, the role of PacC in regulating development and pathogenicity has been demonstrated in a variety of pathogenic fungi, such as *F. graminearum*, *Magnaporthe oryzae*,* B. cinerea*, *Penicillium expansum*, *S. sclerotiorum* and the human pathogenic fungus *Candida albicans* [3, 20–24]. However, the interactions between the Pal/PacC pathway components and the specific mechanisms by which they regulate virulence in fungal pathogens remain unclear.

*Fusarium graminearum* is the primary fungus responsible for causing *Fusarium* head blight (FHB) in various cereals, including wheat, barley, maize, and other agricultural crops globally [25–27]. Additionally, infected grains become contaminated with mycotoxins such as zearalenone (ZEN) and deoxynivalenol (DON), resulting in significant issues such as severe effects on food production and mycotoxin contamination [28–30]. In our previous study, we reported that *F. graminearum* has the ability to induce host alkalinization during *F.* graminearum–wheat interactions. FgPacC30 (a 30-kDa active form of FgPacC) subsequently plays a crucial role in fungal adaptation to host-derived high-iron stress and successful infection, revealing the molecular mechanisms by which pathogenic fungi resist environmental iron stress at the epigenetic level [20]. In this study, by using homologous recombination to construct targeted deletions of Pal/PacC pathway components in *F. graminearum*, we found that mutants of the Pal/PacC pathway components exhibited significant growth defects, except for ΔFgPalI, as well as a significant increase in sensitivity to cationic, oxidative, cytoplasmic membrane and osmotic stresses. The western blot analysis revealed that FgPacC, a key element in the Pal/PacC pathway, could be cleaved to the functional isoform FgPacC30 after alkaline pH was reached or, independently, by NaCl-induced osmotic stress. Further studies elucidated the direct interactions between the essential components of this signaling pathway in *F. graminearum* for the first time and revealed that ambient pH-dependent proteolytic activation regulates the subcellular localization of FgPacC. More importantly, we revealed that FgPacC30 inhibits the activity of the histone acetyltransferase FgGcn5, thus transcriptionally downregulating the expression of the *FgTRI1* gene and inhibiting DON biosynthesis. Overall, this study highlights the critical role of the pH regulatory system in the response to external stress and virulence in *F. graminearum*, providing a new perspective for understanding the pathogenic mechanisms in phytopathogenic fungi.

## Materials and methods

### Fungal strains and culture conditions

The wild-type strain PH-1 of *F. graminearum* (NRRL 31084) was used as the parental strain for this study [31]. The mycelia of both the wild-type PH-1 strain and the other derived strains were cultured on minimum medium (MM), potato dextrose agar (PDA), or complete medium (CM) at 25 °C [32]. The mycelial radial growth of each strain was measured via a caliper gauge at two perpendicular diameters, and the average diameter for each plate was computed following the method described by Weitz et al. [33]. Conidiation was induced in carboxymethylcellulose sodium (CMC) spore-producing liquid media. For conidial germination assays, conidial suspensions of 5 × 10^5^ conidia/mL were incubated on yeast extract peptone dextrose (YEPD) liquid media (20 g of glucose, 3 g of yeast extract and 10 g of peptone) at 25 °C. The germination rate and germ tube length were measured via a light microscope (ZEISS PrimoStar). For sexual reproduction, each strain was inoculated and incubated on carrot agar as previously described [34]. Each experiment was independently replicated three times.

### Mutant generation and complementation

Targeted gene replacement was carried out via the polyethylene glycol (PEG)-mediated protoplast transformation technique via a hygromycin resistance cassette as previously described [35]. Table S1 shows the primers used for gene replacement and mutant identification. The deletion mutants were subsequently validated through polymerase chain reaction (PCR) (Fig. S1). To complement the deletion mutants, we cotransformed the XK1-25 yeast strain with both the complete gene fragment containing its natural promoter and the *Xho*I-linearized pYF11 plasmid [20]. Each mutant protoplast was subsequently transformed with this construct. PCR validation was conducted to confirm successful transformation (Fig. S1). All the generated mutant strains were preserved in 15% glycerol solution at −80 °C for long-term storage.

### Electrophoretic mobility shift assay (EMSA)

To determine the binding affinity of FgPacC30 for the target promoter, we purified and eluted the FgPacC30-His protein according to previously described methods [20] (Fig. S2). Biotinylated DNA probes were synthesized by GenScript (Nanjing, China). An electrophoretic mobility shift assay (EMSA) was performed via a LightShift™ Chemiluminescent EMSA Kit (Thermo Fisher, USA) according to the manufacturer's protocol [36]. Briefly, biotin-labeled probes were combined with purified FgPacC30-His and allowed to incubate at ambient temperature for 20 min. The resulting complexes were then electrophoresed through a 6% polyacrylamide gel for 90 min before being blotted onto a positively charged nylon membrane (Millipore, USA). Detection was performed via an Image Quant LAS4000 mini imaging system (GE Healthcare, USA).

### Yeast two-hybrid (Y2H)

For Y2H plasmid construction, gene-specific primers (Table S1) were used to amplify coding sequences from wild-type cDNA. The amplified fragments were subsequently cloned and inserted into either the pGADT7 or pGBKT7 vector. These constructs were then introduced into *S. cerevisiae* AH109 via LiAc/SS-DNA/PEG-mediated transformation [32]. The standard controls included positive (pGBKT7-53 + pGADT7-T) and negative (pGBKT7-Lam + pGADT7-T) interaction pairs. The transformants were grown for 3‒5 days on synthetic medium (SD) lacking Leu and Trp at 30 °C and then transferred to SD medium without His, Leu or Trp, but 3 mM 3-AT (3-amino-1,2,4-triazole) was added to evaluate protein‒protein interactions. Each Y2H result was confirmed by three independent experiments.

### Microscopy Observation

To construct a GFP-FgPacC fusion cassette, the GFP fragment was first amplified from the vector, and the FgPacC open-reading fragment was amplified from wild-type genomic DNA. The resulting PCR products were fused by PCR and co-transformed with *Xho*I-digested pYF11 into the yeast strain XK1-25 as described previously [31]. Finally, the GFP fluorescence of fresh mycelia and conidia was observed under a Zeiss LSM780 confocal microscope (Carl Zeiss AG, Germany). For observation of the nucleus, freshly harvested mycelia were stained simultaneously with 4′,6-Diamidino-2-phenylindole dihydrochloride (Sigma-Aldrich, USA).

### DON production assays

Mycotoxin production was assessed by culturing strains either in trichothecene biosynthesis induction (TBI) medium or on wheat kernels. Following extraction and purification, DON was determined through LC–MS/MS (Liquid Chromatography-Tandem Mass Spectrometry) system according to established protocols [37, 38].

### RNA extraction and RT‒qPCR assays

Total RNA was extracted with TRIzol reagent, followed by spectrophotometric quantification. First-strand cDNA synthesis was carried out via the use of 1 µg of total RNA with a PrimeScript™ cDNA synthesis kit (Vazyme Biotech, China). Quantitative PCR was conducted on an ABI QuantStudio™ 5 system (Thermo Fisher) using SYBR Green Master Mix (Vazyme Biotech) with the manufacturer-recommended cycling parameters. Gene expression levels were calculated via the 2^−ΔΔCT^ method, with *FgACTIN* used as the internal control. All primer sequences are provided in Table S1.

### Chromatin immunoprecipitation (ChIP)-qPCR analyses

ChIP was conducted following established procedures with minor adaptations [20]. Briefly, fungal mycelia were cross-linked with 1% formaldehyde for 10 min. Chromatin was fragmented into 200–500 bp fragments via a Covaris E220 sonicator (Woburn, MA). Immunoprecipitation was next performed using anti-H3K18ac (39,755, Active Motif Inc., USA), anti-H2BK11ac (ab240613, Abcam, UK) and protein A agarose beads (sc-2003, Santa Cruz, USA). Following sequential washes [39], complexes were eluted, reverse-crosslinked, and treated with proteinase K. Purified DNA was resuspended in 50 μL ddH₂O after phenol/chloroform extraction. The input DNA was recovered via phenol extraction after the sonication step as a control. The sequences of primers used for ChIP‒qPCR are provided in Table S1. Relative enrichment was calculated as the ratio of the amount of immunoprecipitated DNA to the amount of input DNA.

## Results

### Sequence analysis of the transcription factor FgPacC

To elucidate the function of the Pal/PacC pathway in *F. graminearum*, we first searched for orthologs of *Aspergillus nidulans* PacC, a core component of the Pal/PacC pathway. Using *A. nidulans* PacC as a query, we identified a single PacC ortholog, FGSG_12970 (named FgPacC), from the *F. graminearum* genome via BLASTP. Phylogenetic analysis revealed that FgPacC shares 45% and 51% similarity with homologs of *A. nidulans* and *Neurospora crassa*, respectively (Fig. S3a). Sequence analysis of FgPacC with homologs of *A. nidulans* and *N. crassa* revealed that they have similar protease recognition sites and conserved cleavage regions (Fig. S3b), implying that FgPacC could also generate a functional isoform. To investigate the processing and subcellular localization of FgPacC in *F. graminearum*, we complemented the ΔFgPacC mutant by fusing GFP to the N-terminus of FgPacC under the control of the native promoter of FgPacC. Our previous study revealed that GFP-FgPacC accumulated rapidly in the nucleus only under alkaline conditions [20]. To further explore the external signals that the transcription factor FgPacC responds to, we treated the complementary strain ΔFgPacC::GFP-FgPacC under alkaline or NaCl conditions. The western blot analysis revealed that GFP-FgPacC was cleaved into a 30-kDa active form (named FgPacC30 hereafter) under 1 M NaCl conditions in addition to alkaline pH conditions (Fig. S3c-d), indicating that the Pal/PacC pathway can respond to several environmental signals.

### The FgPal/FgPacC pathway is required for fungal growth in *F. graminearum*

**Fig. 1:**
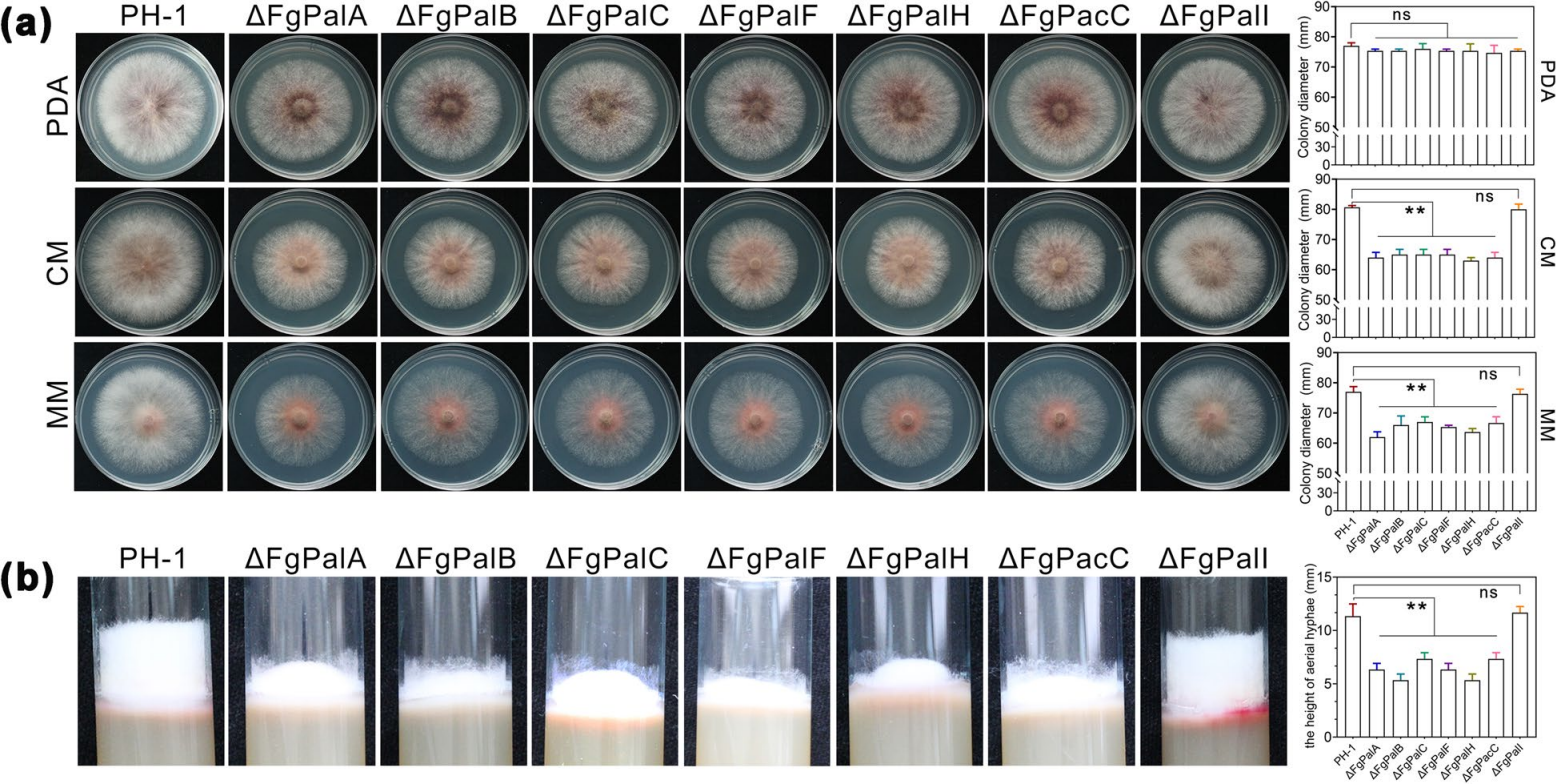
The FgPal/FgPacC pathway affects the mycelial growth of *F. graminearum*. **a** Growth of wild-type PH-1 and the knockout mutants ΔFgPalA, ΔFgPalB, ΔFgPalC, ΔFgPalF, ΔFgPalH, ΔFgPacC, and ΔFgPalI on PDA, CM, and MM media. A 5 mm mycelial plug of each strain was incubated for three days on each medium. Subsequently, photographs were captured, and the colony diameter of each strain was measured. The data are presented as the means ± SD (ns: not significant, **p < 0.01, Student’s *t-*test, *n* = 3). **b** Wild-type PH-1 and knockout mutants were cultured on potato dextrose agar (PDA medium) in test tubes for three days to observe and measure the height of the aerial hyphae of each strain visually. The data are presented as the means ± SD (ns: not significant, **p < 0.01, Student’s *t-*test, *n* = 3)

Referring to the FgPacC search method, we further searched for other genes in the Pal/PacC pathway in *F. graminearum*, including FgPalA, FgPalB, FgPalC, FgPalF, FgPalH, and FgPalI, and found that both FgPalH and FgPalI are transmembrane proteins (Fig. S4). We next generated deletion mutants via a homologous recombination strategy and named them ΔFgPalA, ΔFgPalB, ΔFgPalC, ΔFgPalF, ΔFgPalH, and ΔFgPalI (Fig. S1). To characterize the function of the Pal/PacC pathway in mycelial growth, each strain was cultured on potato dextrose agar (PDA), complete medium (CM), or minimal medium (MM). Phenotypic analysis revealed that every single Pal/PacC pathway component deficiency, with the exception of ΔFgPalI, impaired *F. graminearum* hyphal growth compared with that of the wild-type PH-1 on PDA, CM and MM (Fig. 1a-b). Although the colony diameters of the mutants ΔFgPalA, ΔFgPalB, ΔFgPalC, ΔFgPalF, ΔFgPalH, and ΔFgPacC did not appear to be significantly different from that of the wild-type strain on PDA plates (Fig. 1a), the height of the aerial hyphae of these mutants was notably reduced in tube culture (Fig. 1b). In addition, we constructed the complementary mutants ΔFgPalA-C, ΔFgPalB-C, ΔFgPalC-C, ΔFgPalF-C, ΔFgPalH-C, ΔFgPacC-C and ΔFgPalI-C. The growth of the complemented mutants was similar to that of the wild type in the selected media (Fig. S5). With respect to sexual and asexual reproduction, the FgPal/FgPacC pathway did not affect spore germination or perithecium formation (Table S2 and Fig. S6). Therefore, the FgPal/FgPacC pathway is crucial for fungal growth but is not essential for sexual or asexual reproduction in *F. graminearum*.

### The FgPal/FgPacC pathway is involved in the response to multiple stresses

**Fig. 2:**
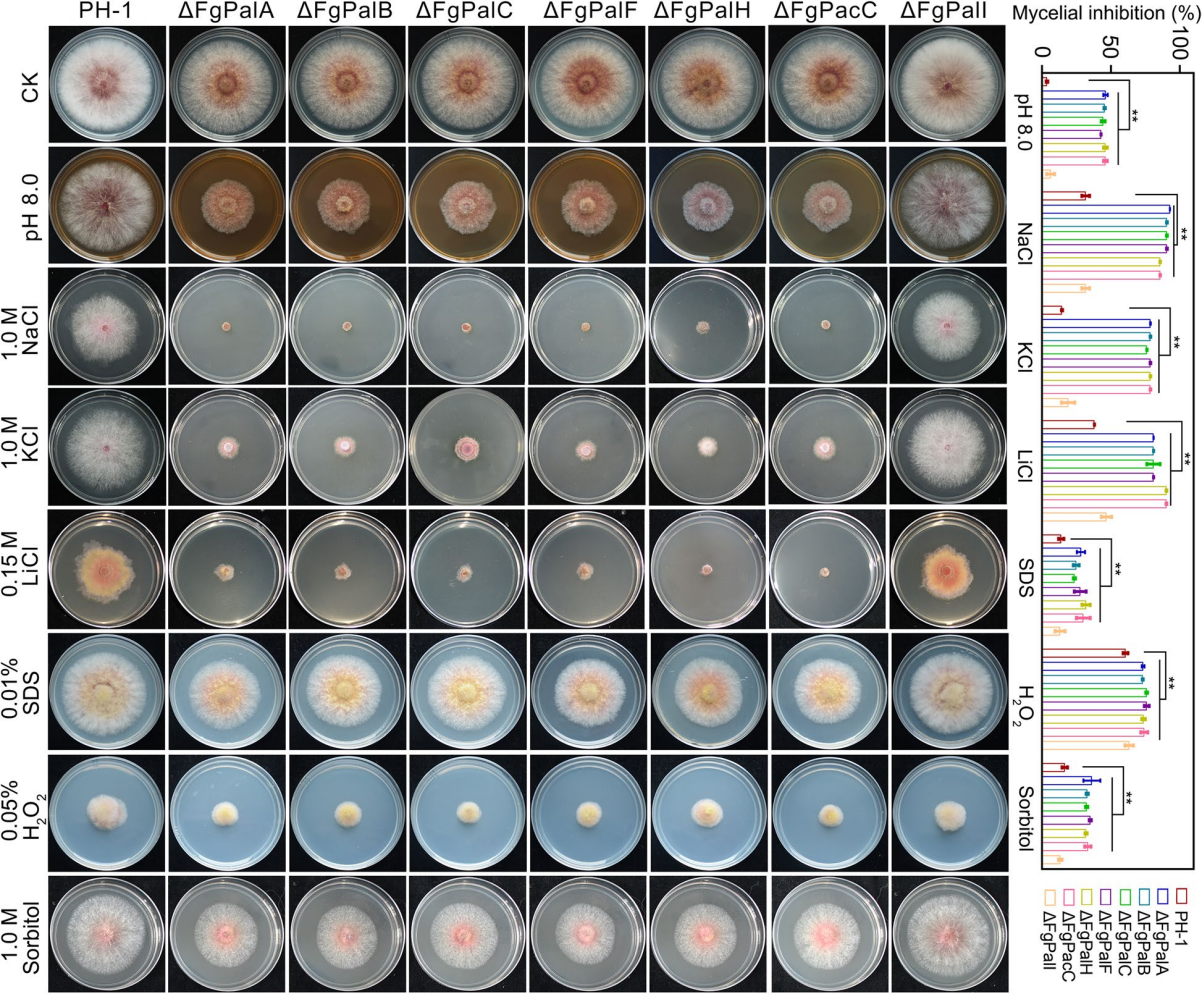
The FgPal/FgPacC pathway influences multiple stress responses in *F. graminearum*. The growth of wild-type PH-1 and the knockout mutants ΔFgPalA, ΔFgPalB, ΔFgPalC, ΔFgPalF, ΔFgPalH, ΔFgPacC, and ΔFgPalI on PDA with or without pH 8.0, 1.0 M NaCl, 1.0 M KCl, 0.15 M LiCl, 1.0 M sorbitol or 0.01% SDS and 0.05% H_2_O_2_. Mycelial inhibition by each stress condition relative to the untreated control was calculated for each strain. The data are presented as the means ± SD (**p < 0.01, Student’s *t-*test, n = 3)

To sustain their growth and reproduction, fungi must adapt to a variety of complex stresses under natural conditions. We first examined the sensitivity of wild-type PH-1 and every single FgPal/FgPacC pathway component mutant to alkaline pH. As indicated in Fig. 2, the mycelial growth of ΔFgPalA, ΔFgPalB, ΔFgPalC, ΔFgPalF, ΔFgPalH and ΔFgPacC was significantly inhibited on pH 8.0 PDA medium compared with that of the wild-type PH-1 strain. However, ΔFgPalI was insensitive to alkaline pH, suggesting that FgPalI is not involved in the response to alkaline conditions. Furthermore, we observed that these mutants also exhibited greatly increased sensitivity to osmotic stresses [1.0 M NaCl, 1.0 M KCl, 0.15 M LiCl, 1.0 M sorbitol or 0.01% SDS (sodium dodecyl sulfate)] and oxidative stress (0.05% H_2_O_2_) (Fig. 2), indicating the crucial role of the FgPal/FgPacC pathway in mediating tolerance to various environmental stresses.

### Ambient pH affects fungal penetration via the FgPal/FgPacC pathway during infection

**Fig. 3:**
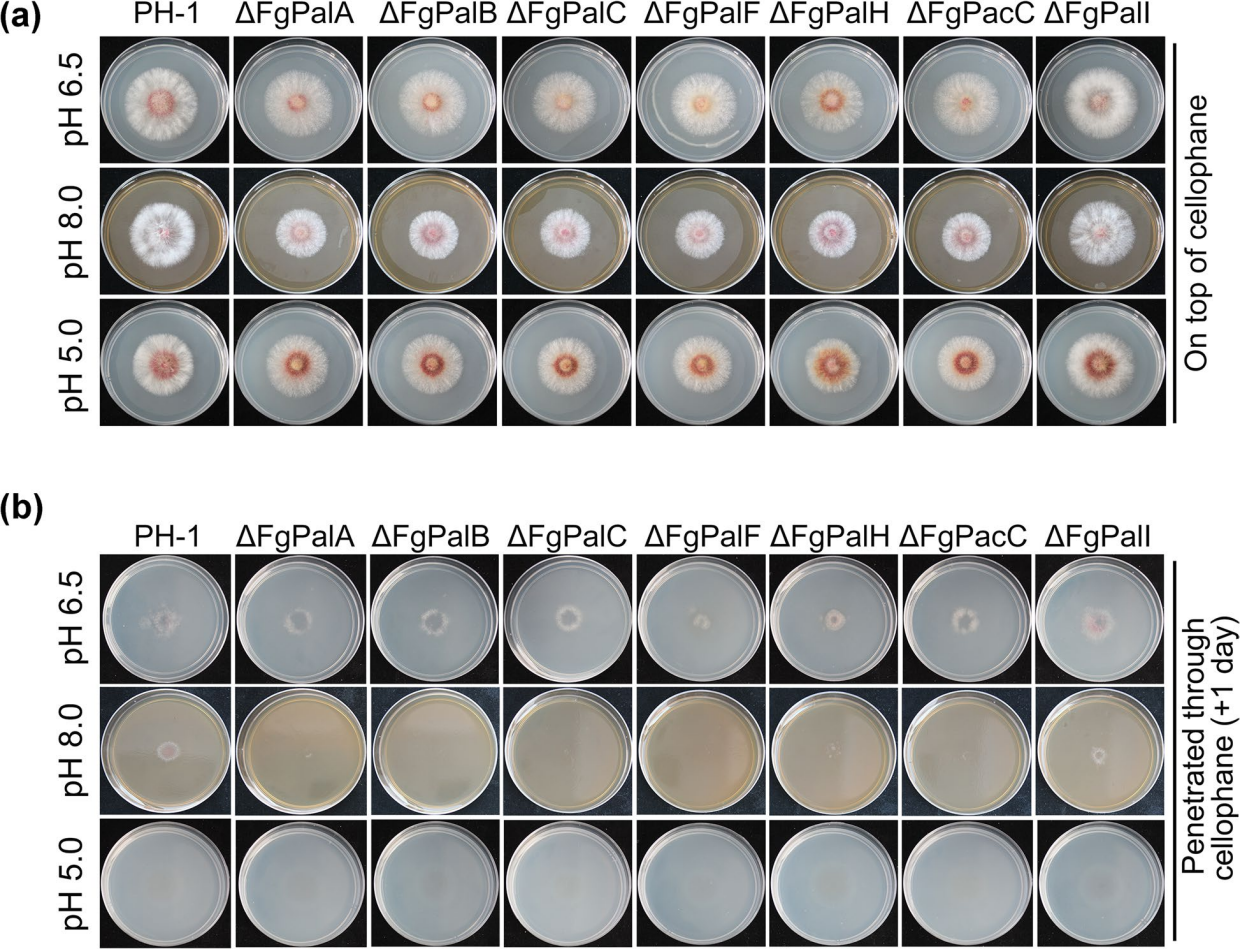
FgPal/FgPacC pathway affects the mycelial penetration of *F. graminearum* under alkaline conditions. Each strain was grown for 2 days on pH 6.5, pH 8.0 and pH 4.0 PDA medium covered with a single layer of cellophane (**a**), after which the strains were cultured for 1 day after the cellophane was removed (**b**)

Our previous study demonstrated that *F. graminearum* infection is able to induce rapid extracellular alkalinization in wheat tissues [20]. To evaluate the virulence function of the FgPal/FgPacC pathway during the host-alkalization process, we next examined the growth of strains on acidic, neutral or alkaline PDA medium covered with a single layer of cellophane. As shown in Fig. 3a, the fungi absorbed nutrients through cellophane and grew normally on cellophane, but the mutants ΔFgPalA, ΔFgPalB, ΔFgPalC, ΔFgPalF, ΔFgPalH, and ΔFgPacC were significantly more inhibited after 2 days of growth under alkaline conditions than under neutral or acidic conditions. More importantly, we removed the cellophane and cultured the strains for 1 day. The FgPalA, FgPalB, FgPalC, FgPalF, FgPalH and FgPacC mutants failed to penetrate the cellophane membranes under alkaline pH conditions (Fig. 3b), indicating that the ambient pH affects fungal penetration via the FgPal/FgPacC pathway. In addition, neither the wild type nor the mutants were able to penetrate under acidic pH conditions (Fig. 3b), suggesting that the acidic host environment does not affect the growth of *F. graminearum* but directly inhibits the penetration ability of the fungus.

### Interactions between key components of the FgPal/FgPacC pathway

**Fig. 4:**
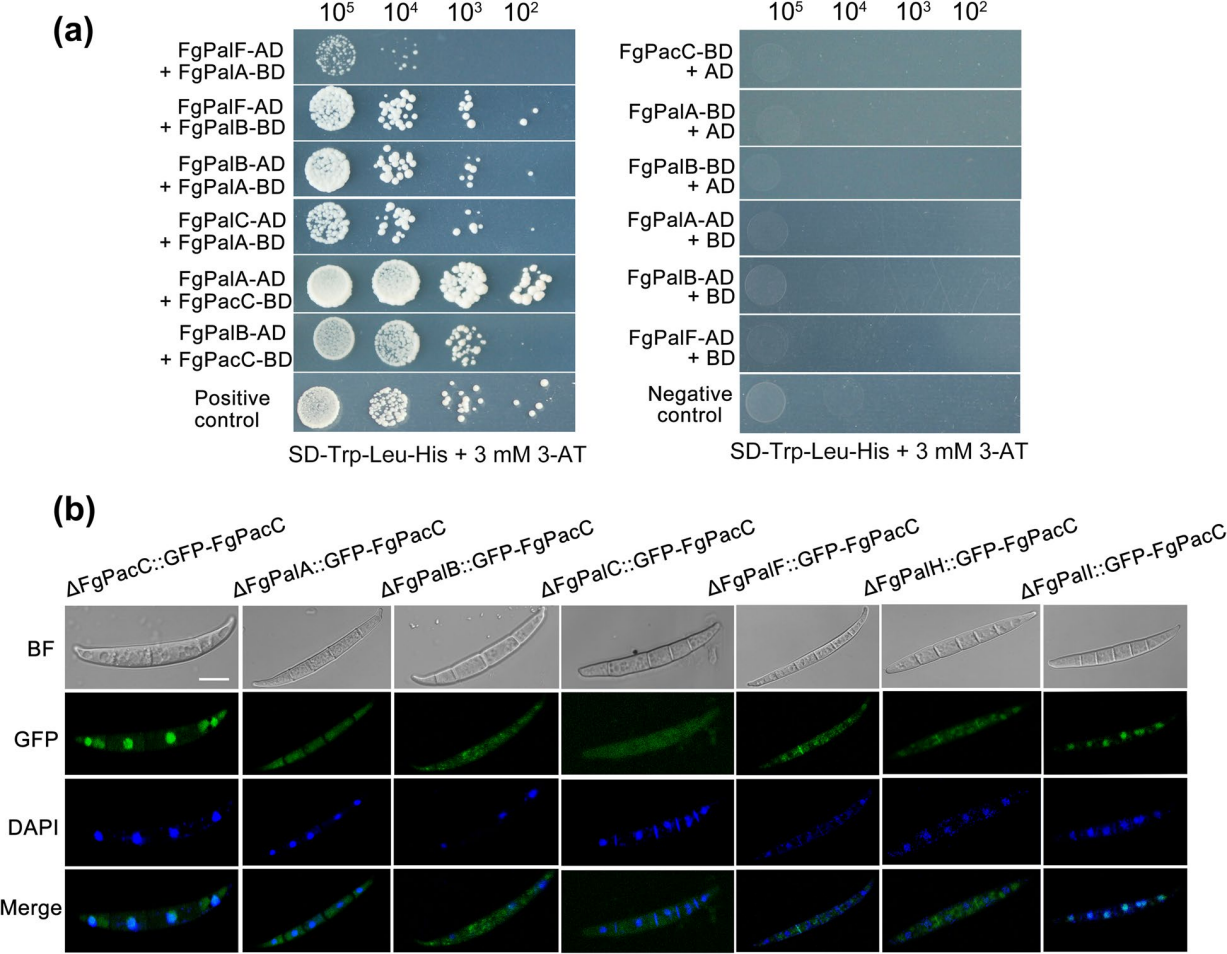
Relationships between upstream FgPal proteins and the transcription factor FgPacC. **a** Yeast cells transformed with the FgPal/FgPacC pathway component-prey and the FgPal/FgPacC pathway component-bait were serially diluted and cultured on synthetic defined (SD) medium lacking leucine (L), tryptophan (T), and histidine (H) but supplemented with 3 mM 3-AT (3-amino-1,2,4-triazole). **b** Subcellular localization of GFP-FgPacC in the ΔFgPacC, ΔFgPalA, ΔFgPalB, ΔFgPalC, ΔFgPalF, ΔFgPalH or ΔFgPalI mutants at alkaline pH. Nuclear staining with DAPI was also performed to confirm nuclear localization. Scale bar = 10 μm

In *A. nidulans*, the pH-signal-transduction process involving the Pal/PacC pathway has been well studied [2]. We further performed Y2H assays to examine the interactions between the key components of the FgPal/FgPacC pathway in *F. graminearum*. As indicated in Fig. 4a, FgPalF, a protein downstream of the plasma membrane complex, interacts with FgPalA and the cysteine protease FgPalB, whereas FgPalA interacts with FgPalB and FgPalC. Furthermore, both FgPalA and FgPalB were found to bind with the transcription factor FgPacC. These interactions suggest the existence of a complex network within the FgPal/FgPacC pathway, which dynamically regulates fungal adaptability and interacts with environmental signals.

To further analyze the relationship between upstream FgPal proteins and FgPacC, we used confocal epifluorescence microscopy and found that GFP-FgPacC rapidly translocated into the nucleus under alkaline pH conditions, but GFP signals diffused into the cytoplasm in the ΔFgPalA, ΔFgPalB, ΔFgPalC, ΔFgPalF, and ΔFgPalH mutants, but not in the ΔFgPalI strain (Fig. 4b). Taken together, these findings suggest that ambient pH-dependent proteolytic activation regulates the subcellular localization of FgPacC.

### FgPacC negatively regulates DON production by reducing FgGcn5-mediated histone acetylation in *F. graminearum*

**Fig. 5:**
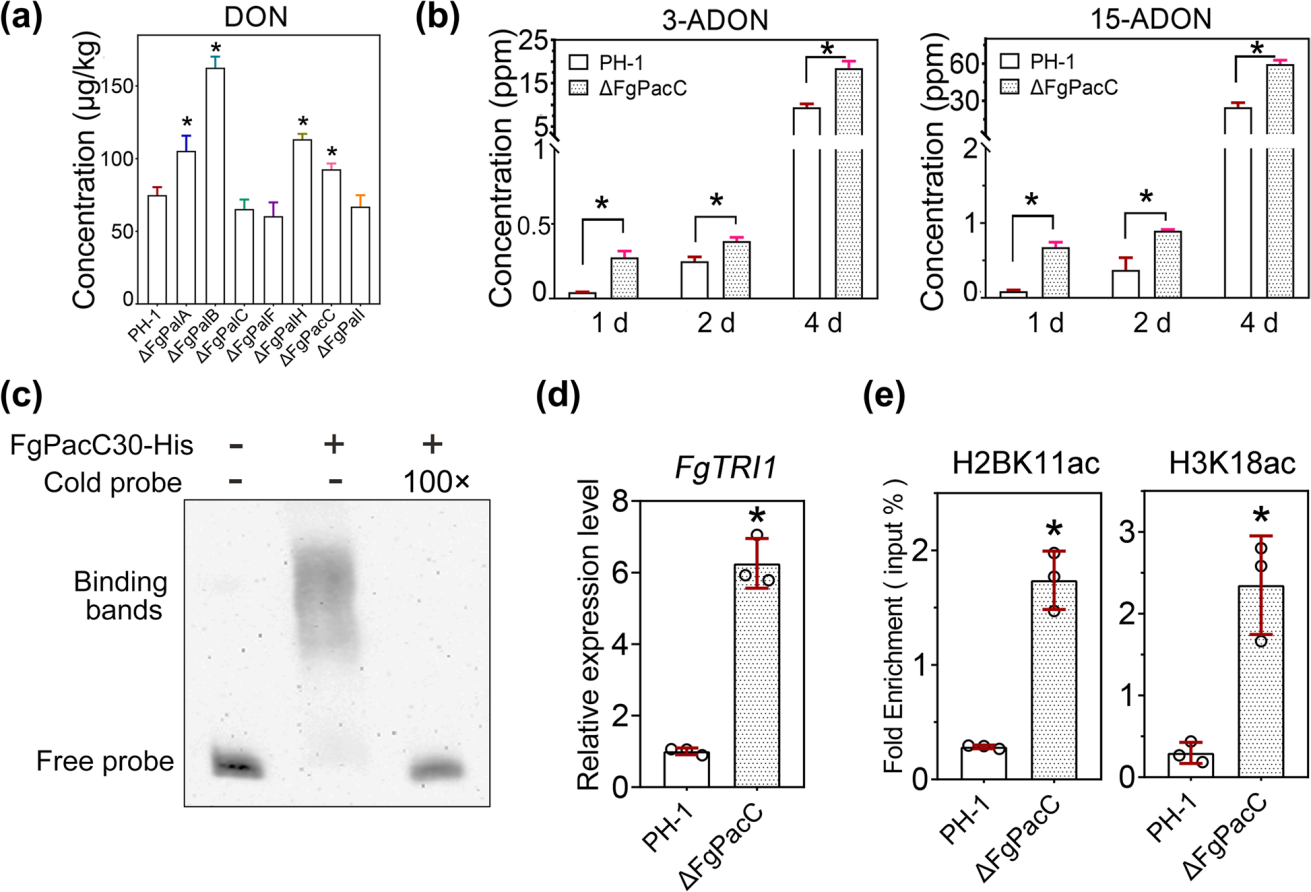
FgPacC directly binds to *FgTRI1* and negatively regulates its expression by inhibiting the acetylation of H3K18 and H2BK11. **a** DON production was measured in wheat kernels inoculated with PH-1 and its derived mutants (ΔFgPalA-I). The statistical significance of the difference compared with that of the wild-type PH-1 strain was determined by Student's *t*-test (*p* < 0.05). The data are the means ± SD (*n* = 3). **b** 3-ADON and 15-ADON were measured for wild-type PH-1 and ΔFgPacC after they were cultured for 1, 2 and 4 days in TBI medium. The data are presented as the means ± SD (*p* < 0.05, Student’s *t-*test, *n* = 3). **c** EMSA confirmed the binding of FgPacC30 to the *FgTRI1* promoter region. Binding specificity was verified through competition experiments using a 100-fold excess of unlabeled DNA fragment. **d** Under alkaline conditions, RT‒PCR was performed to detect *FgTRI1* expression levels in both wild-type PH-1 and ΔFgPacC, with *FgACTIN* serving as the internal reference. The statistical significance of the differences compared with that of the wild-type PH-1 strain was assessed via Student's *t*-test (*p* < 0.05). The data are the means ± SD (*n* = 3). **e** ChIP‒qPCR demonstrated the deposition of H3K18ac and H2BK11ac at the *FgTRI1* promoter in PH-1 and ΔFgPacC under alkaline conditions. The ChIP- and input- DNA samples were quantified via quantitative PCR. The statistical significance of the differences compared with that of the wild-type PH-1 strain was assessed via Student's *t*-test (*p* < 0.05). The data are the means ± SD (*n* = 3)

DON is an essential virulence factor for *F. graminearum* [40, 41]. We first examined the production of DON in wheat kernels infected with wild-type PH-1 and FgPal/FgPacC pathway mutants. As shown in Fig. 5a, ΔFgPacC, ΔFgPalA, ΔFgPalB, and ΔFgPalH all exhibited significantly increased DON production. We further assayed the production of 3-ADON and 15-ADON, the precursors of DON, after 1, 2 or 4 days of incubation in trichothecene biosynthesis induction (TBI) medium and found that FgPacC negatively regulates 3-ADON and 15-ADON biosynthesis (Fig. 5b). To clarify the molecular mechanism by which FgPacC30 inhibits DON production, we first conducted EMSA, and the results revealed that FgPacC30 directly binds to the promoter of the DON biosynthetic enzyme *FgTRI1* (Fig. 5c). RT‒qPCR confirmed that the expression of *FgTRI1* was significantly increased in ΔFgPacC under alkaline conditions (Fig. 5d). Our previous study revealed that FgPacC30 represses iron uptake gene expression by inhibiting the histone acetyltransferase activity of FgGcn5 on H3K18 and H2BK11 [20]. Accordingly, we compared the H2BK11ac and H3K18ac signals in the region of *FgTRI1* in wild-type PH-1 and ΔFgPacC via ChIP‒qPCR. As shown in Fig. 5e, under alkaline conditions, the enrichment of H2BK11ac and H3K18ac in the *FgTRI1* promoter in ΔFgPacC was significantly greater than that in PH-1, which further suggests that FgPacC30 represses gene expression by influencing the epigenetic mechanism. Collectively, these findings suggest that the absence of FgPacC leads to a relaxation of the chromatin state at the *FgTRI1* promoter, thereby increasing its expression and contributing to elevated DON production in the fungus.

## Discussion

**Fig. 6:**
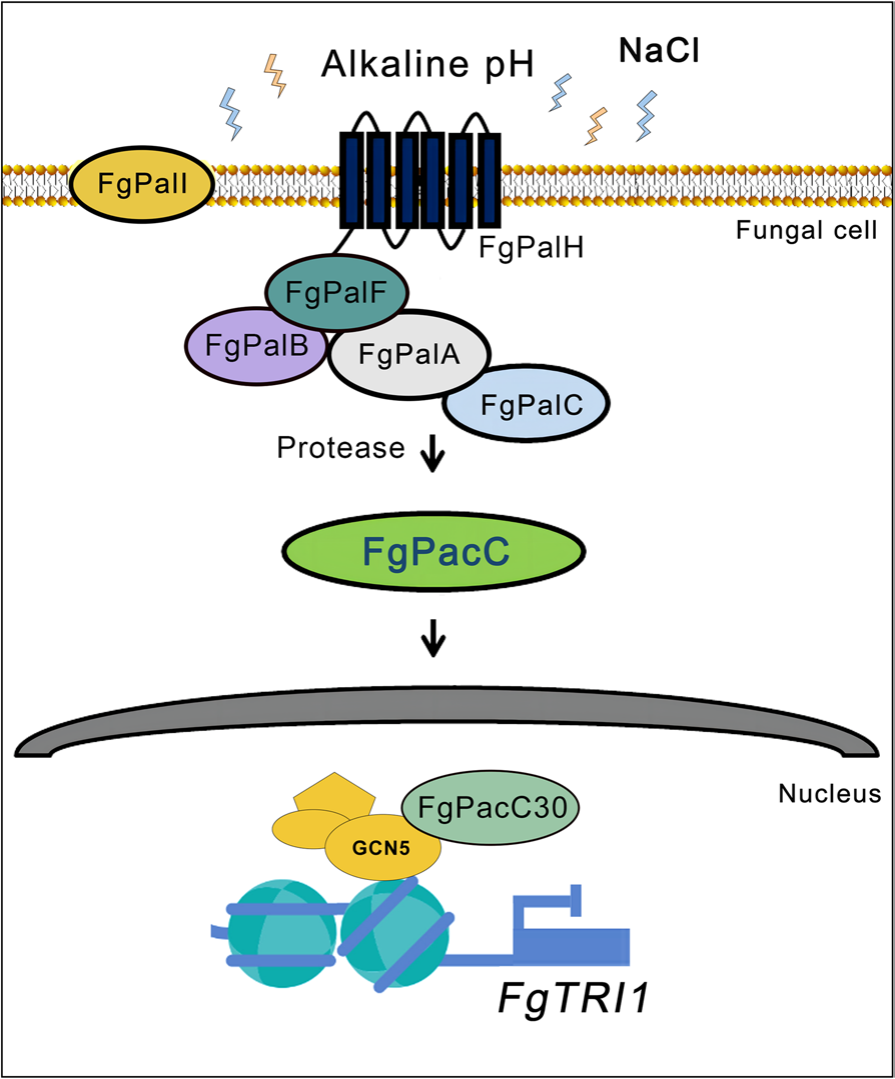
A proposed model for the FgPal/FgPacC signaling pathway and epigenetic negative regulation of *FgTRI1* in *F. graminearum*. External signals, such as alkaline or NaCl conditions, ultimately cleave FgPacC from the full-length protein to the 30-kDa active form via the upstream FgPal pathway. The functional isoform FgPacC30 plays a transcriptional inhibitory role after being transferred to the nucleus. Specifically, FgPacC30 deactivates H3K18ac and H2BK11ac in the *FgTRI1* promoter region, inhibiting *FgTRI1* expression and thereby negatively regulating DON production

Alkalinity and salinity not only affect soil quality but also serve as important environmental stress factors that plant pathogenic fungi, such as *F. graminearum* and *F. oxysporum*, need to overcome [12, 42–44]. The balance of acid–base is significant to maintain normal physiological integrity and cellular responses. Fungi have developed a conserved Pal/Rim signaling pathway that responds to alkaline pH, and it was first described in the ascomycete fungi *A. nidulans* and *S. cerevisiae* [2]. In *A. nidulans*, the transmembrane protein PalH can capture alkaline pH signals and influence the ubiquitination and phosphorylation of PalF. This process leads to the activation and processing of the zinc finger transcription factor PacC through the proteasome, which involves PalA, PalB and PalC. Ultimately, the shortened PacC is translocated into the nucleus, where it functions as a transcriptional regulator [2, 19]. Similar mechanisms involving the Pal/PacC signaling pathway have also been reported in various fungal pathogens, including *F. oxysporum*, *C. neoformans* and *Yarrowia lipolytica*, indicating that the pH signaling system is widely conserved across the fungal kingdom [45–47]. In our study, we identified the FgPal/FgPacC pathway in *F. graminearum* for the first time and investigated the interactions between key components of the FgPal/FgPacC pathway, further elucidating its complex regulatory network (Fig. 6). Understanding these interactions can provide insights into how *F. graminearum* integrates signals from its environment to fine-tune its growth and pathogenicity. Salinity stress alters membrane integrity, enzyme activity, and protein and nucleic acid metabolism by affecting the osmotic homeostasis of living cells [48–52]. Our results suggest that in addition to alkaline pH, NaCl stress can also trigger proteolytic cleavage of FgPacC into the functional isoform FgPacC30. Furthermore, our findings indicate that the FgPal/FgPacC signaling pathway is essential not only for fungal growth but also for the ability of fungi to adapt to various environments, including several salt stresses. Moreover, the ambient pH influences fungal penetration ability during infection, suggesting that the FgPal/FgPacC pathway is critical for the adaptability of *F. graminearum.*

Deoxynivalenol (DON), a critical secondary metabolite produced by the phytopathogen *F. graminearum* complex, can lead to serious crop diseases and health risks [53, 54]. In addition to its direct biological toxicity, DON has also been identified as a significant virulence factor for *F. graminearum*, playing an important role in the dissemination of the fungus within host tissues [55–57]. Chromatin-mediated epigenetic modifications have a significant effect on trichothecene biosynthesis in *F. graminearum* [58–62]*.* The methyltransferase FgSet1 controls the enrichment of histone 3 lysine 4 di- and trimethylation (H3K4me2/3) on FgTRIs, thereby promoting the production of DON [58]. However, the deletion of the putative H3K27 methyltransferase KMT6 results in the constitutive expression of genes associated with mycotoxins and other secondary metabolites [61]. To date, histone acetyltransferases such as Gcn5, Sas3, Fng1 and Elp3 have been reported to play regulatory roles in the induction of DON [62–65]. These findings collectively suggest that epigenetic regulation is crucial for controlling the production of DON. In addition, acidic pH is important for promoting *TRI* gene transcription and trichothecene production in *F. graminearum* in vitro [66], but our previous studies indicated that *F. graminearum* infection increases the pH of nearly 3 units from acidic levels in wheat tissue, leading to alkalinization of the host plant [20]. Further studies revealed that, during infection, *F*. *graminearum* has the capacity to generate substantial quantities of DON in wheat heads and little DON during vegetative growth [56, 67]. However, the underlying mechanism by which *F. graminearum* inhibits DON toxin production during the growth of invasive mycelia is still unclear. In this study, we found that FgPacC serves as a negative regulator of DON production through the reduction of FgGcn5-mediated histone acetylation. Mechanistically, *F. graminearum* provoked plant alkalinization in wheat tissues, triggering FgPacC30 activation and direct binding to FgGcn5, which inhibited its HAT activity, thus transcriptionally down-regulating the expression of the *FgTRI1* gene and inhibiting DON biosynthesis (Fig. 6). To the best of our knowledge, this is the first report of a mechanism of DON biosynthesis inhibition by a transcription factor-mediated SAGA complex that represses the transcription of the *TRI* gene at the epigenetic level.

In conclusion, our study highlights FgPacC as a crucial regulator in *F. graminearum*, orchestrating responses to environmental stresses and mediating virulence through the FgPal/FgPacC pathway. The regulatory duality of FgPacC positions it as a central regulator in *F. graminearum*. Future work should elucidate the Pal/PacC regulatory network governing fungal pathogenicity and toxigenesis, potentially enabling new control methods for this destructive phytopathogen.

## Supplementary Information

Supplementary Material 1

## Data Availability

The data that support the findings of this study are available within the paper and its supplementary materials.
