# Supplementary material for: The pH signaling pathway Pal/PacC regulates fungal growth, stress responses, and mycotoxin biosynthesis in Fusarium graminearum
Source: Crop Health. 2025 Aug 1;3(1):17. doi: 10.1007/s44297-025-00054-3 (PMC12825994; doi:10.1007/s44297-025-00054-3)

**Supplementary data**

**
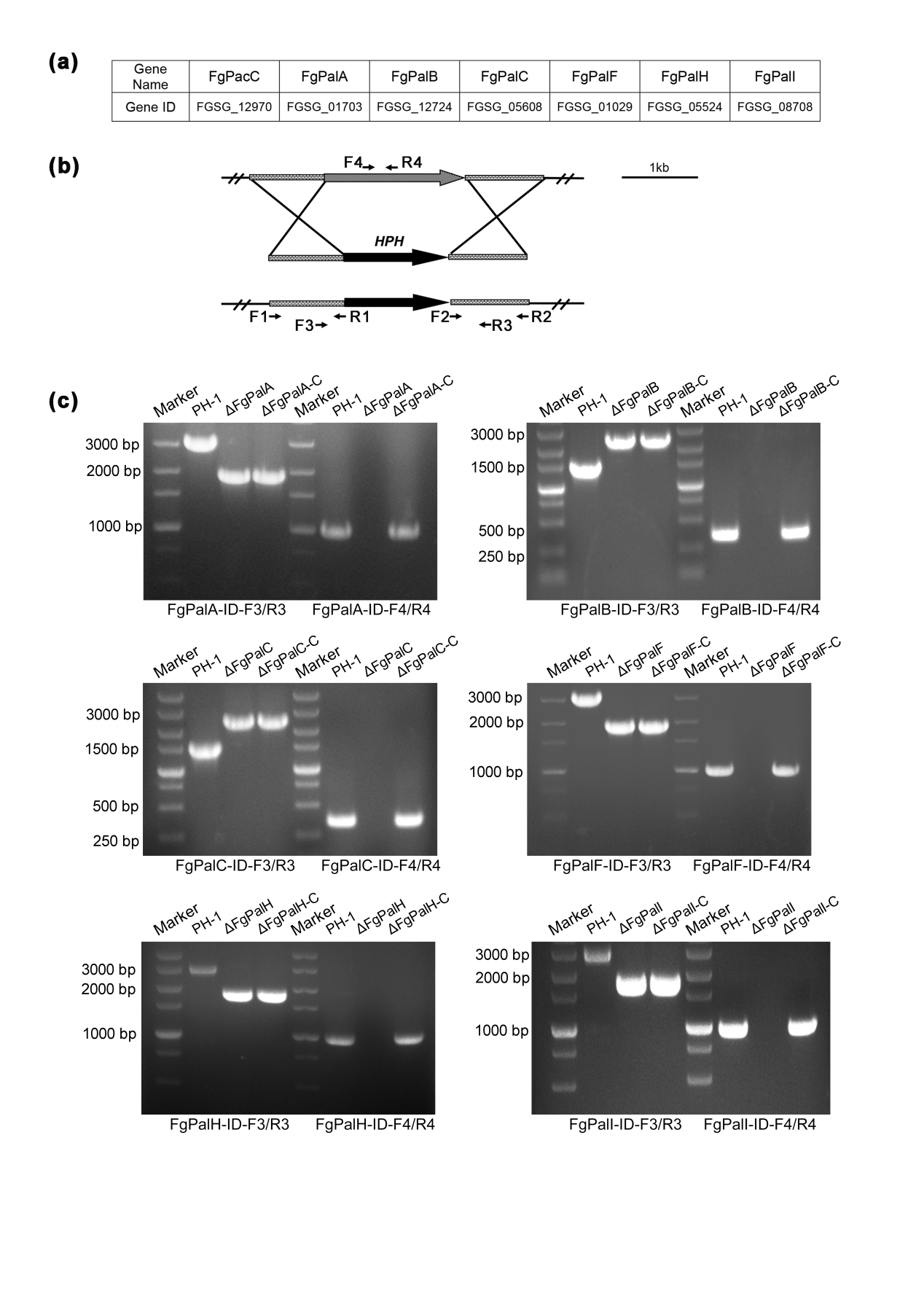
**

Supplementary Figure S1 Construction and identification of gene deletion and complemented mutants. (a) Gene ID of each FgPal/FgPacC pathway compent in *F. graminearum*. (b) Gene replacement strategy for each deletion mutant ΔFgPalA, ΔFgPalB, ΔFgPalC, ΔFgPalF, ΔFgPalH and ΔFgPalI (ΔFgPacC have been obtained in previous studies). (c) PCR verification assays of the wild-type PH-1, each deletion mutant and each complemented strain were performed by using a pair of primers outside each gene (F3+R3) and inside the gene ( F4+R4 ), respectively.


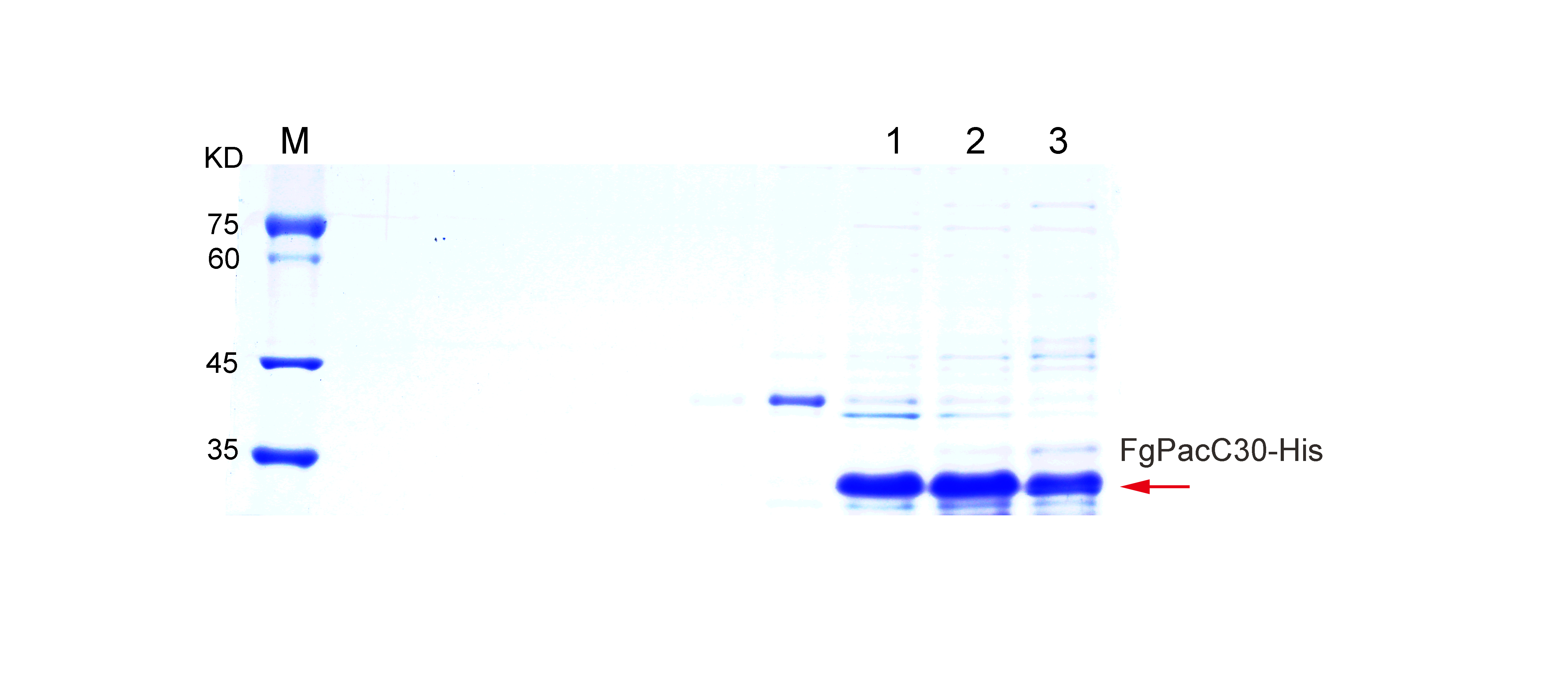


Supplementary Figure S2 Expression and purification of His-tagged FgPacC30 protein. Fusion proteins containing a 6×His tag were expressed in *E. coli* BL21 upon induction for 12 hours at 16 °C in the presence of 0.5 mM isopropyl-β-d-thiogalactopyranoside, and then purified by using Ni-NTA affinity chromatography. Coomassie Brilliant Blue G-250 staining showed the protein samples resolved by SDS-PAGE.


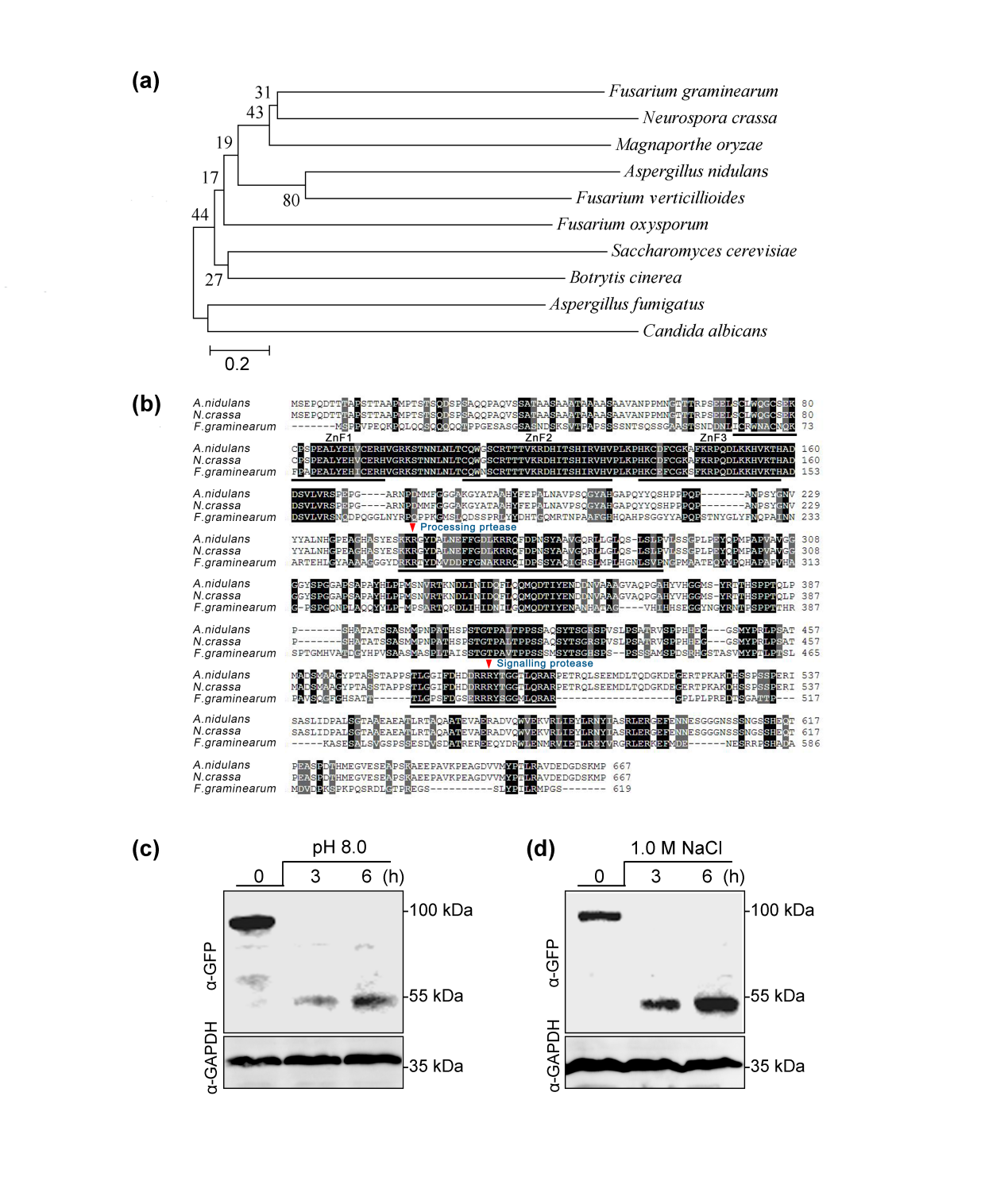


Supplementary Figure S3 (a) Phylogenetic analysis of amino acid sequences of PacC from *F. graminearum* and other fungi, including *Aspergillus nidulans*, *Saccharomyces cerevisiae*, *Aspergillus fumigatus*, *Neurospora crassa*, *Botrytis cinerea*, *Candida albicans*, *Magnaporthe oryzae*, *Fusarium oxysporum* and *Fusarium verticillioides.* (b) Alignment of FgPacC with the homologous amino acid sequences in *A. nidulans* and *N. crassa.* Typical sequences including C2H2-type zinc fingers domain (ZnF1, ZnF2 and ZnF3), processing protease site and signalling protease site have been indicated. (c-d) Western blot analysis of N-terminal GFP-tagged FgPacC (GFP-FgPacC) processing in wild-type PH-1 grown shifted to alkaline (OH^-^) and 1.0 M NaCl conditions for the different time points.


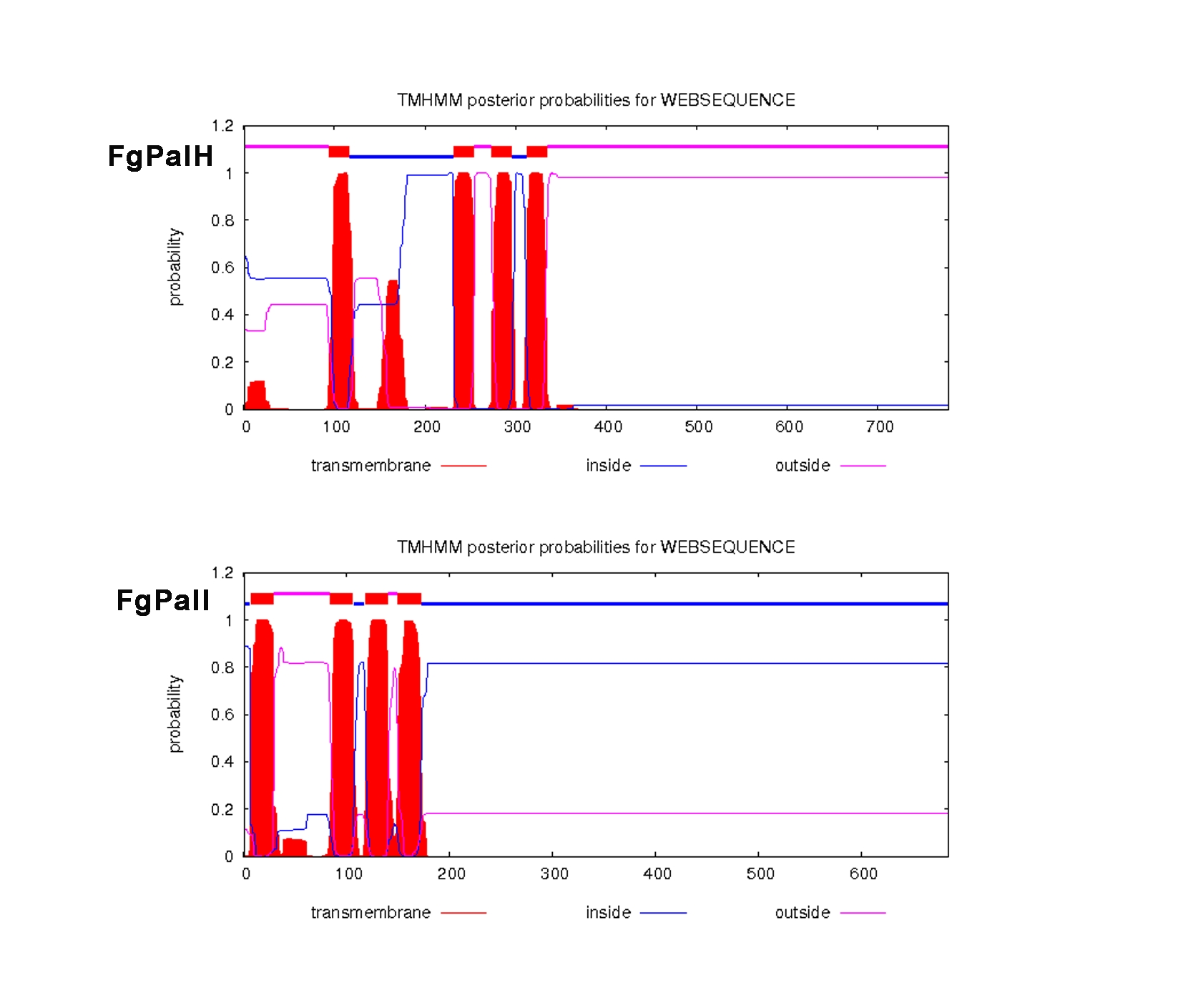


Supplementary Figure S4 TMHMM-2.0 was used to predict protein transmembrane helice in FgPalH and FgPalI.


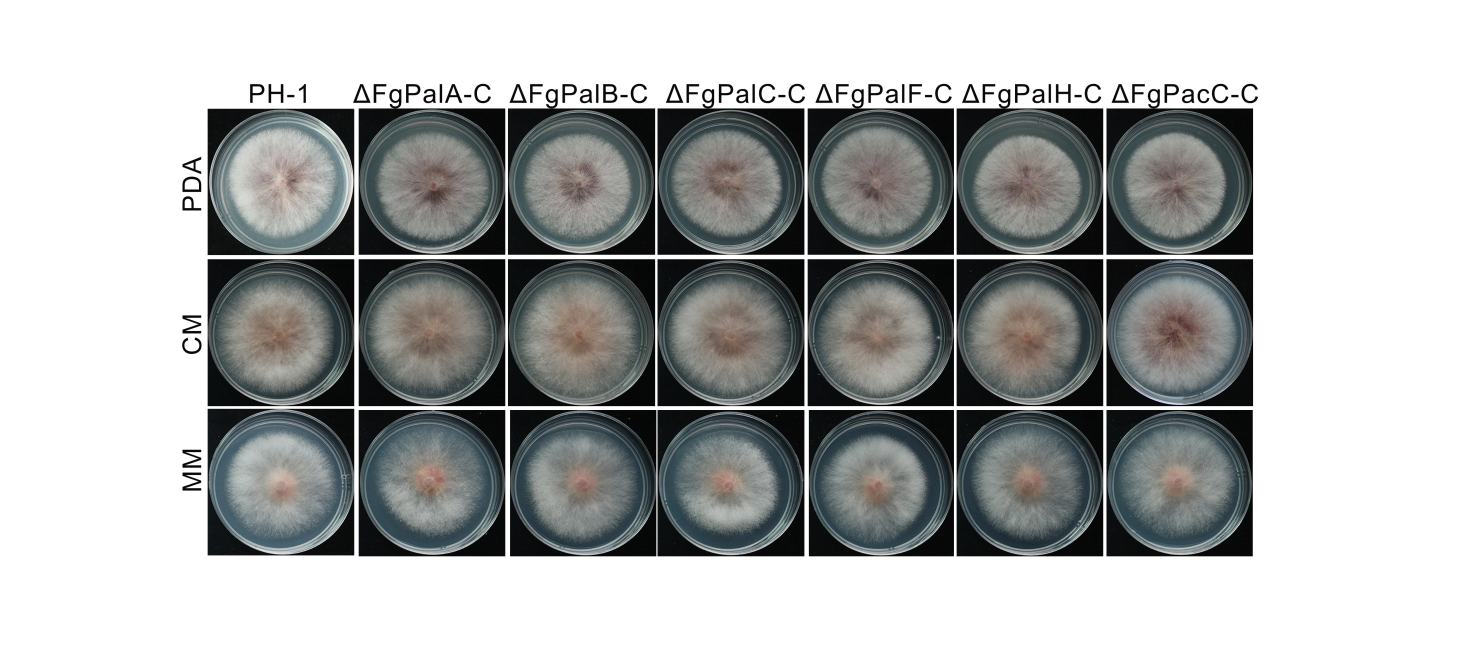


Supplementary Figure S5 The growth of wild-type PH-1 and complementary mutants ΔFgPalA-C, ΔFgPalB-C, ΔFgPalC-C, ΔFgPalF-C, ΔFgPalH-C, ΔFgPacC-C, and ΔFgPalI-C on PDA, CM, and MM media. A five-mm mycelial plug of each strain was incubated for three days on each medium.


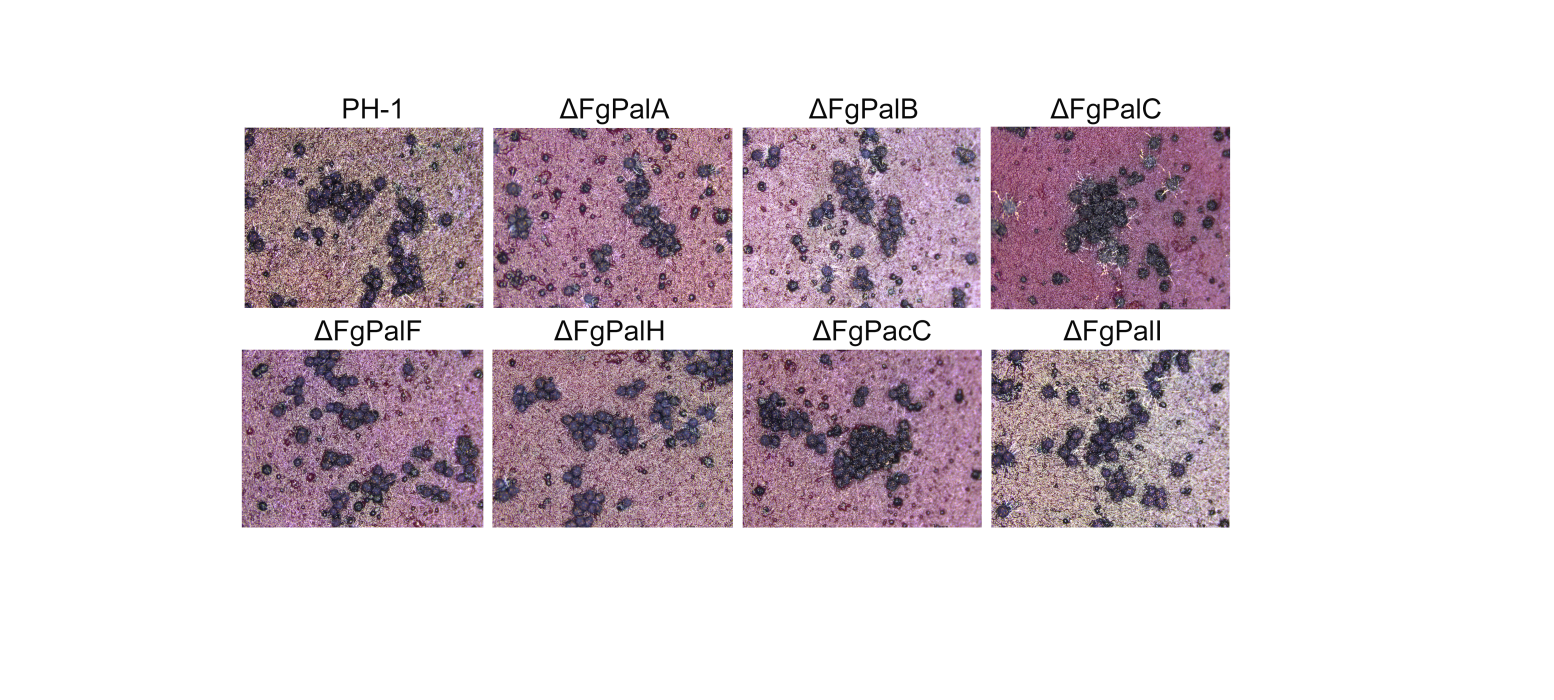


Supplementary Figure S6 Conidia of wild-type PH-1 and knockout mutant ΔFgPalA, ΔFgPalB, ΔFgPalC, ΔFgPalF, ΔFgPalH, ΔFgPacC, and ΔFgPalI were inoculated onto carrot agar for 20 days, and the aerial hyphae were removed for clear visualization of clusters of perithecia formed on the agar surface.

**Supplementary Table S1 Primers used in this study**

| **Name** | **Sequence (5′-3′)** |
| --- | --- |
| FgPalA-HPH-up-F1 | GTTGCTCTTGGCAGTGATCT |
| FgPalA-HPH-up-R1 | CAAAATAGGCATTGATGTGTTGACCTCCAGTCACAAGTTATCAAGCCG |
| FgPalA-HPH-down-F2 | CTCGTCCGAGGGCAAAGGAATAGAGTAGTATATCTGGCGAACTGTCAT |
| FgPalA-HPH-down-R2 | CGAGAGCCACTCCAAGCAAG |
| FgPalA-ID-F3 | GTGAATAATCCTCTCTCGTC |
| FgPalA-ID-R3 | ACGCAGCAGTGATGATGATG |
| FgPalA-ID-F4 | TCCGCAGCCAACTATTTCTC |
| FgPalA-ID-R4 | ATCCCCTAATCGCAGTTTGT |
| FgPalB-HPH-up-F1 | TCCTGTGGCGGCCATAAAAC |
| FgPalB-HPH-up-R1 | CAAAATAGGCATTGATGTGTTGACCTCCCAAGGTTTGCCAAGGGAGAC |
| FgPalB-HPH-down-F2 | CTCGTCCGAGGGCAAAGGAATAGAGTAG ACGGTCCATCTCTACTGGAC |
| FgPalB-HPH-down-R2 | TCTGTAATAATGGTTGCTCC |
| FgPalB-ID-F3 | CACGGTCCACAATTGATGAG |
| FgPalB-ID-R3 | ATTGTGCGGCCATTTGGAGC |
| FgPalB-ID-F4 | ATCACCATGGACTCTCGACG |
| FgPalB-ID-R4 | TATTGGAGAACGCTTTGATG |
| FgPalC-HPH-up-F1 | TTGAAGTCTGCTGTATCGTC |
| FgPalC-HPH-up-R1 | CAAAATAGGCATTGATGTGTTGACCTCCAATGCTTTCTGTCCGTGGTT |
| FgPalC-HPH-down-F2 | CTCGTCCGAGGGCAAAGGAATAGAGTAG CTCGGAACATGCAGCCAAAG |
| FgPalC-HPH-down-R2 | ATCACTGGAGATGGGGAATT |
| FgPalC-ID-F3 | TATCCCCACAAACCAGCAGC |
| FgPalC-ID-R3 | AACCCACCCAATACCGTCTG |
| FgPalC-ID-F4 | AAAGCACAAAAGACTCCCAC |
| FgPalC-ID-R4 | ACTTTGTCGCTGTCTGGATT |
| FgPalF-HPH-up-F1 | :TTTCATCTCAACTCGCCCAG |
| FgPalF-HPH-up-R1 | CAAAATAGGCATTGATGTGTTGACCTCCAAGCACACGCACATACCCAT |
| **Name** | **Sequence (5′-3′)** |
| FgPalF-HPH-down-F2 | CTCGTCCGAGGGCAAAGGAATAGAGTAG GTTGGCATTTCGCCTTTGTC |
| FgPalF-HPH-down-R2 | CGGTGACGGCATTCGCTTAT |
| FgPalF-ID-F3 | CACGGCATCTGCCACCTCTT |
| FgPalF-ID-R3 | TGCTGAGAAGGAAGAGGATC |
| FgPalF-ID-F4 | CGAAACCGAAGGAGAAAGTC |
| FgPalF-ID-R4 | TATCCTCTTCGTCGCTTTCG |
| FgPalH-HPH-up-F1 | TCTCTGCCTGATTGTTCCAC |
| FgPalH-HPH-up-R1 | CAAAATAGGCATTGATGTGTTGACCTCCCTGGCCCGTTGTTCAGTTAG |
| FgPalH-HPH-down-F2 | CTCGTCCGAGGGCAAAGGAATAGAGTAG TTTGTGTGCTGATAGTTGTG |
| FgPalH-HPH-down-R2 | TTGTGCGGGATCGTTGCTTG |
| FgPalH-ID-F3 | TCCCAGTTTCCCCCCACGAT |
| FgPalH-ID-R3 | TCTAAAACAACTAAACGGCC |
| FgPalH-ID-F4 | CTTTCCCAATCTGTTACGCC |
| FgPalH-ID-R4 | TGGCAATAGAAGAGACAGTG |
| FgPalI-HPH-up-F1 | TTATTGGCAACCTCCCCAAG |
| FgPalI-HPH-up-R1 | CAAAATAGGCATTGATGTGTTGACCTCCCGCTTGGTAGAAAACTGATG |
| FgPalI-HPH-down-F2 | CTCGTCCGAGGGCAAAGGAATAGAGTAGGCTATTTGCCCAAGAGACAC |
| FgPalI-HPH-down-R2 | TGAATCTGCCGAGTAGGCCT |
| FgPalI-ID-F3 | TCGCATCTTCACCCATTCAC |
| FgPalI-ID-R3 | TACAATACCCTCTCCCTCTC |
| FgPalI-ID-F4 | TCAGCAGGAAAGACAGGCAG |
| FgPalI-ID-R4 | TGCGTGCGTGCGTATCATAC |
| FgPalA-C-F | TTTCGTAGGAACCCAATCTTCAAAATGGCGTCGTAAGCTCCAA |
| FgPalA-C-R | CACCACCCCGGTGAACAGCTCCTCGCCCTTGCTCACGCCAAACTTGATCCCAGCGT |
| FgPalC-C-F | TTTCGTAGGAACCCAATCTTCAAAATGCCGTTTCCTTTCGTTCT |
| FgPalC-C-R | CACCACCCCGGTGAACAGCTCCTCGCCCTTGCTCACATAGTATGCATTGCCTGGCGT |
| FgPalF-C-F | TTTCGTAGGAACCCAATCTTCAAAATGGTTACAGCGACGCTAACG |
| **Name** | **Sequence (5′-3′)** |
| FgPalF-C-R | CACCACCCCGGTGAACAGCTCCTCGCCCTTGCTCACTCTTTGGTACGCAGGTAACTG |
| FgPalH-C-F | TTTCGTAGGAACCCAATCTTCAAAATGACCGCCGCCGGCTTGATC |
| FgPalH-C-R | CACCACCCCGGTGAACAGCTCCTCGCCCTTGCTCACGGTCGCTCGGCGGGTTCCCG |
| FgPacC-BD-F | atggccatggaggccgaattcATGTCTCCTCCAGTTCCTGAGCA |
| FgPacC-BD-R | tcgacggatccccgggaattcCTAAGAGCCAGGCATGCGAA |
| FgPalA-AD-F | gccatggaggccagtgaattcATGGCGTCGTAAGCTCCAGA |
| FgPalA-AD-R | atgcccacccgggtggaattcTTAGCCAAACTTGATCCCAGCG |
| FgPalA-BD-F | atggccatggaggccgaattcATGGCGTCGTAAGCTCCAGA |
| FgPalA-BD-R | tcgacggatccccgggaattcTTAGCCAAACTTGATCCCAGCG |
| FgPalB-AD-F | gccatggaggccagtgaattcATGGAGAAGAGAGCTCAGGTAAGTCA |
| FgPalB-AD-R | atgcccacccgggtggaattcTTACGTAAAAGGTTCCTCTCCTGG |
| FgPalB-BD-F | atggccatggaggccgaattcATGGAGAAGAGAGCTCAGGTAAGTCA |
| FgPalB-BD-R | tcgacggatccccgggaattcTTACGTAAAAGGTTCCTCTCCTGG |
| FgPalC-AD-F | gccatggaggccagtgaattcATGCCGTTTCCTTTCGTTCTT |
| FgPalC-AD-R | atgcccacccgggtggaattcCTAATAGTATGCATTGCCTGGCG |
| FgPalF-AD-F | gccatggaggccagtgaattcATGGTTACAGCGACGCTAACG |
| FgPalF-AD-R | atgcccacccgggtggaattcTTATCTTTGGTACGCAGGTAACTGC |
| FgTRI1-EMSA-F | AGTCGGTCTATGAGTCTGTG |
| FgTRI1-EMSA-R | GACAGCGAAATGGTCTGTCAAG |
| FgTRI1-qPCR-F | CAGGAGAGTTATTGCCGAAG |
| FgTRI1-qPCR-R | CTGAGAGTGTGATGTCGGAC |
| FgTRI1-CHIP-F | GAAACCACGGCTGAGTATCG |
| FgTRI1-CHIP-R | GGTATCGTTATCTTCACATG |

**Supplementary Table S2 Spore germination rate and length of germ tube in wild type and knockout mutants under neutral conditions**

| **Strain** | **Spore germination rate (100%)** | | **Length of germ tube (μm)** | |
| --- | --- | --- | --- | --- |
|  | **3h** | **6h** | **3h** | **6h** |
| PH-1 | 0.68±0.03a | 0.86±0.02a | 6.27±0.09a | 75.89±1.32a |
| ΔFgPalA | 0.66±0.02a | 0.864±0.01a | 6.19±0.09a | 74.95±0.63a |
| ΔFgPalB | 0.6±0.03a | 0.87±0.03a | 6.06±0.07a | 73.52±2.56a |
| ΔFgPalC | 0.66±0.05a | 0.90±0.03a | 6.15±0.08a | 71.76±2.01a |
| ΔFgPalF | 0.63±0.10a | 0.84±0.046a | 6.16±0.06a | 75.06±1.28a |
| ΔFgPalH | 0.66±0.05a | 0.85±0.01a | 6.25±0.08a | 72.66±2.54a |
| ΔFgPacC | 0.59±0.04a | 0.84±0.02a | 6.15±0.06a | 73.62±1.61a |
| ΔFgPalI | 0.64±0.03a | 0.84±0.05a | 6.24±0.04a | 75.42±1.90a |

**WB and gels-Main data**


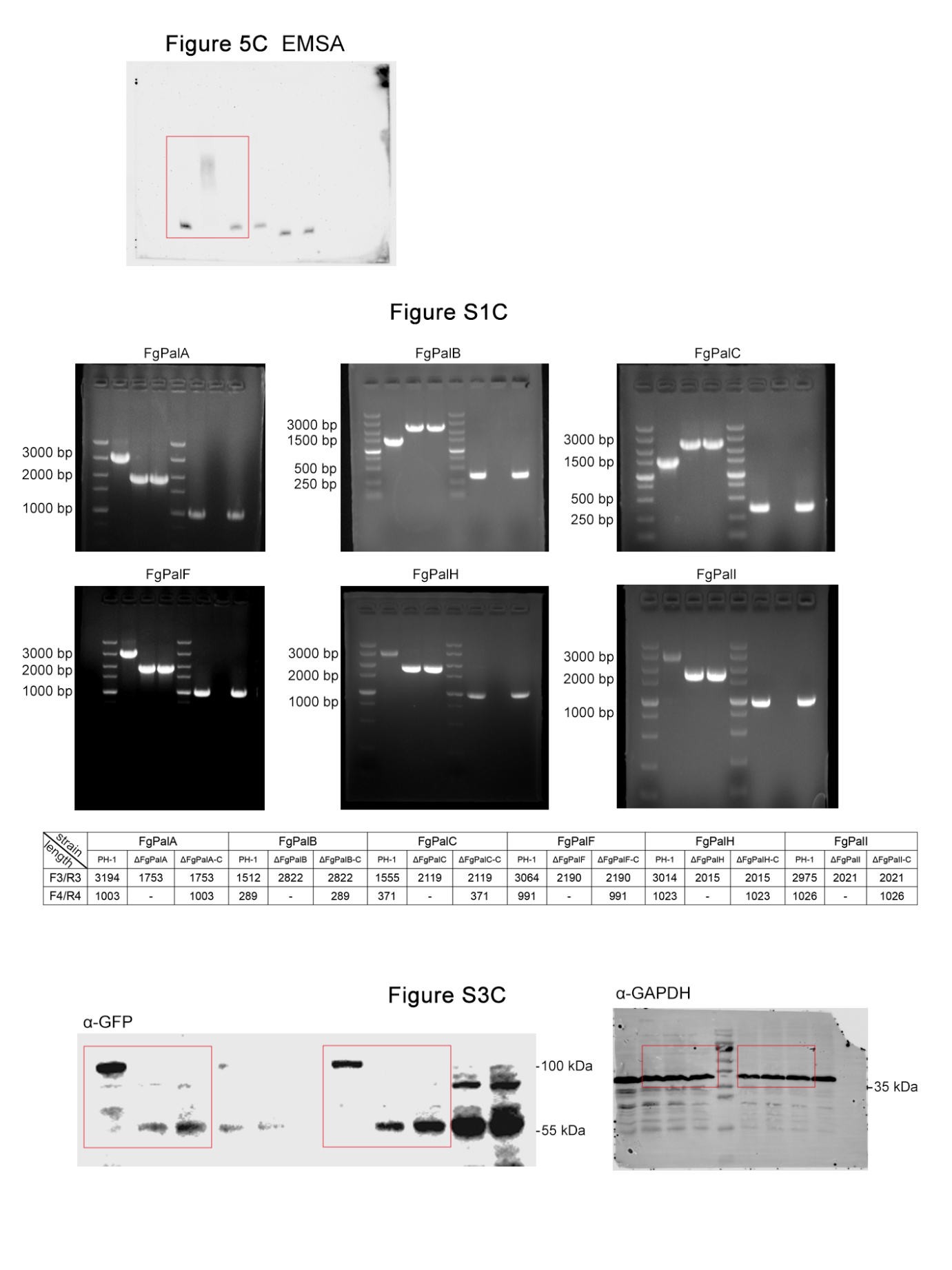

Supplement: Supplementary file 1 — Supplementary Material 1 [file 44297_2025_54_MOESM1_ESM.docx]
